# Adaptive and maladaptive cognitive emotion regulation in child- and adolescent ADHD

**DOI:** 10.1016/j.ijchp.2025.100660

**Published:** 2026-01-08

**Authors:** Rebecka Astenvald, Matilda A. Frick, Johan Lundin Kleberg, Johan Isaksson

**Affiliations:** a Department of Medical Sciences, Child and Adolescent Psychiatry Unit, Uppsala University, Sweden; b Department of Psychology, Stockholm University, Sweden; c Center of Neurodevelopmental Disorders (KIND), Centre for Psychiatry Research, Department of Women’s and Children’s Health, Karolinska Institutet & Stockholm Health Care Services, Region Stockholm, Sweden; d Center for Psychiatry Research, Department of Clinical Neuroscience, Karolinska Institutet (KI) Stockholm, Sweden

**Keywords:** ADHD, Cognitive emotion regulation, Emotion regulation strategies, Comorbidity

## Abstract

**Background:**

Children and adolescents with attention-deficit/hyperactivity disorder (ADHD) often experience difficulties with emotion regulation. Specific use of cognitive emotion regulation strategies may contribute to these challenges, albeit research in this area remain limited.

**Method:**

Self-rated and task-specific use of adaptive and maladaptive cognitive emotion regulation strategies was assessed in children and adolescents with ADHD and typically developing controls (N=176, 10–17 years, 55.1% girls; subsample for self-rated use: N=94, 13–17 years, 61.7% girls). Self-rated use was measured with the short version of the Cognitive Emotion Regulation Questionnaire. Task-specific use was assessed by an experimental task involving viewing emotion-eliciting photos. Regression analyses were utilized to assess associations between ADHD and cognitive emotion regulation. Effects of sex and age were explored. Adjustments were made for co-occurring psychiatric symptoms.

**Results:**

ADHD was associated with lower self-rated (β =-0.21, *p* = .044) and task-specific (β =-0.09, *p* = .015) use of adaptive strategies, and greater use of self-rated maladaptive strategies (β =0.27, *p* = .010). No associations remained after adjusting for co-occurring psychiatric symptoms and multiple comparisons. Rather, depressive symptoms may contribute to the self-rated use of maladaptive strategies. Post-hoc analyses revealed that ADHD was robustly linked to less self-rated use of acceptance (β =-0.38, *p* = .005).

**Conclusion:**

Lower use of self-rated acceptance may be characteristic for ADHD. Depressive symptoms may play a more vital role for maladaptive cognitive emotion regulation than ADHD. More studies are needed to explore the longitudinal relation between ADHD, emotion regulation and depression.

## Introduction

Attention-deficit/hyperactivity disorder (ADHD) is a neurodevelopmental condition characterized by persistent symptoms of inattention and hyperactivity/impulsivity resulting in functional impairment (American Psychiatric Association, 2013). Beyond its core symptomatology, extensive research has demonstrated a robust association between ADHD and emotion regulation challenges (e.g., Beheshti et al., 2020; Graziano & Garcia, 2016). Indeed, 25–45% of children and 70% of adults, with ADHD struggle with emotion dysregulation, which may aggravate psychiatric burden and impairment (Shaw et al., 2014). In addition to regulation, the experiential, behavioural and physiological responses elicited by emotions (i.e., emotional reactivity), interacts with regulatory processes (Gross & Jazaieri, 2014). Thus, more reactive individuals may require stronger or more frequent regulatory efforts to manage their emotional distress. However, our understanding of the specific factors contributing to emotion regulatory challenges remains limited and requires further investigation.

A key aspect of emotion dysregulation concerns difficulties employing effective regulation strategies, a challenge that may span several psychiatric conditions, including ADHD (Gross & Jazaieri, 2014; Sheppes et al., 2015). *Cognitive emotion regulation*, namely utilizing thoughts to regulate emotions during or after experiencing a negative event (Garnefski et al., 2001), may be particularly difficult for individuals with ADHD since the condition is associated with broad cognitive challenges (e.g., Shephard et al., 2022). Specifically, working memory challenges, cognitive flexibility, sustained attention, and cognitive control may constrain the capacity to select, implement, and maintain adaptive emotion regulation strategies (Hendricks & Buchanan, 2016).

A widely used framework to examine cognitive regulation strategies encompasses both *adaptive* and *maladaptive* strategies (Garnefski et al., 2001). Adaptive strategies are often linked to lower psychopathology, whereas maladaptive strategies are associated with higher levels of psychopathology across the lifespan (Aldao et al., 2010; Aldao & Nolen-Hoeksema, 2010; Schäfer et al., 2017). According to Garnefski and colleagues (2001), adaptive strategies comprise acceptance, refocus on planning, positive reappraisal, positive refocusing and putting into perspective, whereas maladaptive strategies include self-blame, other-blame, rumination and catastrophizing. In ADHD, adolescents and young adults have reported greater use of self-blame, rumination, and catastrophizing, and less use of positive reappraisal compared to controls (Mayer et al., 2022) and it has been suggested that a greater reliance on maladaptive strategies may be a characteristic feature of ADHD, although this has mainly been addressed in adults (Alacha et al., 2025; Rüfenacht et al., 2019). Indeed, the only study including youths with ADHD had a small sample size (Mayer et al., 2022) and all of the above-mentioned studies relied solely on self-reports, limiting generalizability.

Cognitive emotion regulation in ADHD has rarely been examined during emotional tasks. The exception regards the use of cognitive reappraisal (i.e., changing the meaning of a stimulus to alter one’s emotional response; Ochsner & Gross, 2008), which is often considered a particularly effective regulation strategy (Augustine & Hemenover, 2009; Webb et al., 2012). While ADHD has been linked to reduced rated use of reappraisal in both children and adults (e.g., Liu et al., 2025; Thorell et al., 2020), children and adolescents with ADHD show similar ability as controls to employ reappraisal during specific tasks (i.e., task-specific use) when receiving instructions (e.g., Hagstrøm et al., 2020; Kiani et al., 2024; Van Cauwenberge et al., 2017). However, the regulation process may be less effective (Liu et al., 2025). Additionally, Matthies and colleagues (2014) found that utilizing acceptance during an emotional task may be an effective regulation strategy for adults with ADHD. To our knowledge, other cognitive regulation strategies have largely been overlooked in experimental tasks involving ADHD. Moreover, the above-mentioned studies only assessed guided emotion regulation, where participants were explicitly instructed to use an emotion regulation strategy. Notably, research suggests potential differences between spontaneous and guided emotion regulation in adults (Ehring et al., 2010), though this remains underexplored in children and adolescents with ADHD.

Childhood and adolescence may be particularly relevant time periods to study emotion regulation, as they entail an increase in negative emotions (Bailen et al., 2019) alongside a transition from reliance on caregivers to the development of internal regulatory skills (Silvers, 2022) and greater use of cognitive regulation (Compas et al., 2017). Conversely, a meta-analysis found no evidence that age moderates emotion regulation in child- and adolescent ADHD (Graziano & Garcia, 2016), albeit they did not account for the use of distinct strategies, which impacted findings in adults (Nolen-Hoeksema & Aldao, 2011). Mixed findings have also emerged, with reports of a positive association between age and reappraisal (Hagstrøm et al., 2020), and no association (Kiani et al., 2024), among youths with ADHD. Together, this highlights the need to consider potential age effects.

Sex differences may also exist in the use of emotion regulation strategies in the general population, both in terms of frequency and type of strategies employed (Lennarz et al., 2019; Nolen-Hoeksema & Aldao, 2011; Smith et al., 2023). In ADHD, a recent review found that adult women rated greater emotion dysregulation compared to men, which may partly be due to reduced access to regulation strategies (Bodalski et al., 2023). Conversely, sex has not been found to moderate emotion regulation in youths with ADHD (Graziano & Garcia, 2016). Potential sex-related effects for cognitive emotion regulation needs clarification.

Considering co-occurring psychiatric symptoms is also important when examining regulation strategies in ADHD since some strategies have been linked to different psychopathologies. For instance, maladaptive strategies have been associated to increased depressive and anxiety symptoms across the lifespan (Aldao et al., 2010; Aldao & Nolen-Hoeksema, 2010; Garnefski et al., 2006; Schäfer et al., 2017), and depression may act as a confounder for the association between ADHD and emotion regulation strategies (Mayer et al., 2022; Rüfenacht et al., 2019). Other commonly co-occurring conditions in ADHD include autism and conduct disorder (CD) (e.g., Franke et al., 2018), both of which are related to emotion dysregulation in children and adults (e.g., Cappadocia et al., 2009; Samson et al., 2014), and may thus serve as potential confounders.

Taken together, it is necessary to employ multi-assessment methods targeting both self-rated and task-specific use of emotion regulation, include younger cohorts with larger sample sizes, and consider possible effects of age, sex, and co-occurring psychiatric symptoms in order to clarify the role of cognitive emotion regulation in child- and adolescent ADHD. The aim of the present study was to examine how self-rated and task-specific use of cognitive emotion regulation strategies may differ between children and adolescents with ADHD and typically developing controls. We hypothesized that ADHD would be related to greater use of maladaptive strategies, and lower use of adaptive strategies, while also exploring potential sex- and age-related effects. Due to sparse research, and some findings suggesting that depression may act as confounder for emotion regulation strategies in older cohorts with ADHD, we adopted an exploratory approach for how co-occurring psychiatric symptoms (i.e., anxiety symptoms, conduct problems and autism traits) may affect the associations.

## Methodology

### Participants and procedure

Children and adolescents (aged 10 to 18 years) with ADHD were recruited from the child- and adolescent psychiatric (CAP) outpatient unit in Uppsala, Sweden, via telephone, post and/or flyers. Inclusion criteria consisted of having a clinical diagnosis of ADHD. Diagnostic status was assessed in accordance with the 5th Edition of the Diagnostic and Statistical Manual (DSM-5) (American Psychiatric Association, 2013) by the CAP unit, where assessments typically involve at least two visits with a clinical psychologist, including medical anamnesis, developmental history and a diagnostic interview. Diagnostic status was further assessed during the study using the ADHD module of the Mini-International Neuropsychiatric Interview for Children and Adolescents (MINI-KID) (Sheehan et al., 2010) in order to determine current symptomatology. Exclusion criteria included co-occurring autism, intellectual disability, psychosis and long-acting ADHD-medication. For the questionnaire data, two participants with ADHD were excluded due to incomplete consent forms and one participant for having an autism diagnosis. For the experimental task data, four participants were excluded for incomplete consent forms and another three due to co-occurring autism. A total of 48%, and 44.9%, of participants with ADHD in the questionnaire data and experimental task data respectively, took ADHD medication, including stimulants (such as methylphenidate) and non-stimulants (such as atomoxetine and guanfacine), according to caregiver reports.

A comparison group of typically developing controls was recruited through the Population Register in Uppsala, where a letter was sent to 1000 families with children aged 10–18 years, inviting them to participate, and via an ad on Meta (i.e., Facebook). As a first step, interested guardians completed a short web-based questionnaire with background questions about the child’s sex, age, confirmation that they do not have a psychiatric diagnosis (which was an exclusion criteria), as well as the parents’ socioeconomic status (SES; education and income). We aimed to match interested families with the children with an ADHD diagnosis based on age and sex. However, due to a limited amount of interested families we were not able to match the samples based on sex. During the study, controls were also screened for ADHD using the MINI-KID (Sheehan et al., 2010). Two (questionnaire data) and seven (experimental task data) participants in the comparison group were excluded for meeting ≥ 6 of the criteria in the inattention or hyperactivity/impulsivity domain for ADHD on the MINI-KID, but lacked information on functional impairment related to these symptoms, preventing further diagnostic evaluation. One participant in the comparison group was relocated to the ADHD group due to 1) screening positively for ADHD on the ADHD module of the MINI-KID (Sheehan et al., 2010) and 2) fulfilling ≥ 6 criteria for ADHD on the parent-rated ADHD Self-Report Scale for adolescents (ASRS-P) (Sjölander et al., 2016).

The final sample comprised 94 participants (53 participants with ADHD) aged 13–17 years for the questionnaire data, assessing self-rated cognitive emotion regulation, and 176 participants (82 participants with ADHD) aged 10–17 years for the experimental task data, assessing task-specific use of cognitive emotion regulation. Note that there is an overlap of participants between both data sets (in total 92 participants). Data was collected from June 2021– March 2025. See Table 1 for sample characteristics.

**Table 1:** Sample characteristics.

	*Questionnaire data (N=94)*			*Experimental task data (N=176)*		
	ADHD n=53	Comparison group n=41	Test statistic	ADHD n=82	Comparison group n=94	Test statistic
Age, median (IQR)	15.00 (3.0)	15.00 (3.0)	Z=1.32	13.00 (3.00)	12.00 (4.00)	Z=1.05
Age range	13–17	13–17		10–17	10–17	
Sex at birth, n (%) females	38 (71.7)	20 (48.8)	χ2=5.14^⁎^	50 (61.0)	47 (50.0)	χ2=2.13
SES a, median (IQR)	3.00 (2.00)	4.00 (1.00)	Z=2.83^⁎⁎^	3.00 (2.00)	4.00 (1.00)	Z=4.01^⁎⁎⁎^
ADHD presentation, n (%) b						
Combined	31 (58.5)			52 (64.2)		
Predominantly inattentive	22 (41.5)			29 (35.8)		
Co-occurring psychiatric conditions, n (%) c						
Anxiety conditions	13 (24.5)	0	χ2=11.67^⁎⁎⁎^	16 (19.5)	0	χ2=18.34^⁎⁎⁎^
Depressive conditions	9 (17.0)	0	χ2=7.70^⁎⁎^	8 (9.8)	0	χ2=8.71^⁎⁎^
Impulsive/disruptive conditions	3 (5.7)	0	χ2=2.40	4 (4.9)	0	χ2=4.25^⁎^
Other neurodevelopmental conditions	6 (11.3)	1 (2.4)	χ2=2.65	13 (15.9)	3 (3.2)	χ2=7.32^⁎⁎^
Other conditions	4 (7.5)	0	χ2=3.23	6 (7.3)	1 (1.1)	χ2=3.92^⁎^
Emotional reactivity, M (SD) d				4.59 (1.54)	4.42 (1.23)	t=0.79

Note: ADHD: Attention deficit/hyperactivity disorder, SES: socioeconomic status, IQR: interquartile range; ^⁎^: p <0.05 ^⁎⁎^: p <0.01 ^⁎⁎⁎^: p <0.001

Three caregiver ratings for the questionnaire data, and 13 caregiver ratings (four were missing for adolescents with ADHD and nine for adolescents in the comparison group) on other psychiatric symptoms were excluded due to complete missing data. In cases where contingency tables included cells with zero counts, p-value from Fisher’s Exact Test was used instead of the chi-square test.

a Summation of each caregiver’s education level and occupational status

b ADHD presentation was determined by medical diagnosis except for the participant reclassified from the comparison to the ADHD group, whose ADHD presentation was based on the MINI-KID ADHD module. Medical diagnoses were not available for the comparison group

c Co-occurring psychiatric conditions were determined by combining medical diagnosis and caregiver reports for the ADHD group, and caregiver reports only for the comparison group. Examples of co-occurring conditions included: social phobia and generalized anxiety disorder (anxiety conditions); depressive episodes and dysthymia (depressive conditions); oppositional defiant disorder and conduct disorder (impulsive/disruptive conditions); dyslexia and language disorders (other neurodevelopmental conditions) and sleep disorders (other conditions)

d Measured by self-ratings on emotional intensity during the experimental task

Participation included completing self- and caregiver ratings on a digital platform and partaking in various tasks and experiments during a 2.5 hour visit to a research lab in Uppsala. Participants were instructed to refrain from taking their ADHD medication on the same day as the study visit.

Oral and written informed consent were obtained from children, adolescents and their caregiver/s before participation. The participants were compensated with two cinema tickets after participation. The study was approved by the Swedish Ethical Review Authority (Uppsala, Dnr 2020–04,261 and 2022–04,228–01).

### Measures

#### Cognitive emotion regulation

Self-rated cognitive emotion regulation was assessed by the short version of the cognitive emotion regulation questionnaire (CERQ-short) (Garnefski & Kraaij, 2006). The scale consists of 18 items, divided into nine subscales á two items targeting adaptive strategies (i.e., positive refocusing, planning, positive reappraisal, putting into perspective and acceptance) and maladaptive strategies (i.e., self-blame, other-blame, rumination and catastrophizing) (Garnefski & Kraaij, 2006). Items are rated on a scale ranging from 1 ((almost) never) to 5 ((almost) always), where higher scores indicate greater use of a strategy. The total score for adaptive or maladaptive strategies is calculated by summing the scores of the corresponding subscales. The CERQ-short has demonstrated good psychometric properties and has been validated in younger cohorts (Sætren et al., 2024; Santos et al., 2023). The means of the total scores for adaptive and maladaptive strategies were used for the main analyses. Cronbach’s alpha was 0.82 for the former and 0.68 for the latter.

**Figure 1:**
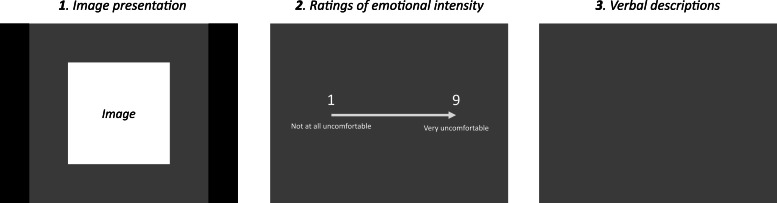
Graphic overview of a photo trial during the experimental task.

*Note*: 1) An image is presented on the computer screen. 2) The participant is asked to rate the intensity of their emotional response to the image 3) The participant is prompted to verbally describe their thoughts about the image

Task-specific cognitive emotion regulation was assessed through an experimental paradigm based on the Reactivity and Regulation-Images task by (Carthy et al., 2010). For this study, only the first part of the task was analysed, wherein participants were instructed to view photos, selected to elicit moderate levels of negative affect, such as threatening animals or catastrophic scenes. A total of 28 photos from the International Affective Picture System, that were rated as negative (i.e., reported valence ≤ 4.3) and moderately distressing (i.e., reported arousal 4.5 to 6.9) in the original study (Lang et al., 2008), were displayed. All photos were transformed to gray-scale colour and converted to achieve equal global luminance using the SaliencyToolbox (Walther & Koch, 2006) in MATLAB. Stimuli were presented on a 1920 × 1080 resolution computer screen using customized scripts developed in Psychtoolbox (Brainard, 1997; Kleiner et al., 2007) in MATLAB. The photos were divided into two sets of 14 photos (*set A*: 1050, 1101, 1201, 1274, 1300, 1930, 2120, 2683, 2810, 6250, 8485, 9050, 9584, 9919 and *set B*: 1111, 1120, 1205, 1271, 1525, 1931, 2130, 2691, 3280, 6260, 9592, 9600, 9611, 9912). Participants were alternatively assigned to view one of the two sets, with the order of presentation randomized within each set. See Figure 1 for a graphic overview of a photo trial.

First, an image was presented on a computer screen for eight seconds. Second, participants were asked to rate the intensity of their emotional response to the image (“How uncomfortable did it feel?”) on a scale from 1 (not at all uncomfortable) to 9 (very uncomfortable). The mean intensity of the emotional response across all 14 photos was used as a measure of emotional reactivity, with a Cronbach’s alpha of 0.82. Third, participants were prompted to describe their thoughts about the image (“Tell me what you thought about the picture”). This instruction could be followed up with a further question (“Did you think of anything else?”). Vocal descriptions were recorded by the test leader using an iPhone. The test leader also recorded the emotional intensity ratings and wrote down summaries of the participant’s descriptions of each photo on a physical test protocol as a backup. Before the task began, two practice images were displayed to familiarize the participant with the procedure.

The vocal descriptions were coded by two assessors into usage of regulation strategies (i.e., 1 [used] or 0 [not used]) corresponding to the strategies in CERQ-short. Cognitive reappraisal was added as an adaptive strategy to capture reinterpretations of the emotion-eliciting stimulus that reduce its negative impact (e.g., “it is not real, it is just a movie”). This differs from positive reappraisal, which involves assigning a positive meaning to the stimulus (e.g., “they will grow stronger from the event”) without changing the meaning of the content. Thought suppression (i.e., blocking thoughts of the negative event; Webb et al., 2012) was added as a maladaptive strategy. Multiple regulation strategies could be coded from the same verbal description. The total use (sum) of adaptive and maladaptive strategies was calculated into two separate variables, which were used for the main analyses. See Supplementary Table 1 for training examples of coding statements and below (“2.3.1 Inter-rater reliability”) for calculations of inter-rater reliability estimates.

#### Co-occurring psychiatric symptoms

Raters for the caregiver reports consisted of 75.8% mothers, 15.4% fathers, 1.1% both parents and 7.7% an unspecified parent in the questionnaire data. For the caregiver reports of co-occurring psychiatric symptoms in the experimental task data, raters were 79.8% mothers, 12.3% fathers, 1.8% both parents, 5.5% an unspecified parent and 0.6% a family home member. Duplicate ratings were found for two caregivers in the comparison group across both data sets. The most recent ratings were chosen for the analyses.

Caregiver ratings of the Montgomery Åsberg Depression Rating Scale (MADRS-P) (Torres Soler et al., 2018) were used to assess depressive symptoms. MADRS-P consist of nine items, which are rated on a scale from 0 to 6, where higher scores indicate more depressive symptoms. MADRS-P has been validated in adolescent samples and demonstrated good psychometric properties (Torres Soler et al., 2018). The mean of the scale was used and Cronbach’s alpha was 0.87 (questionnaire data) and 0.86 (experimental task data).

Caregiver ratings of the Spence Children’s Anxiety Scale (SCAS-P) (Nauta et al., 2004) were used to measure anxiety symptoms. SCAS-P consist of 38 items, rated on a scale from 0 (never) to 3 (always), where higher scores indicate more anxiety symptoms. SCAS-P has demonstrated good psychometric properties (Nauta et al., 2004). The mean of the scale was used and Cronbach’s alpha was 0.95 (questionnaire data) and 0.93 (experimental task data).

Caregiver ratings of the Autism-Tics, AD/HD and other Comorbidities inventory (Hansson et al., 2005) in questionnaire form was used to assess autism traits. This included 17 items, specifically the gate items for language, social interaction and flexibility (Larson et al., 2010). Items are rated on the presence of autism traits using a scale from 0 (no), 0.5 (yes, to some extent) to 1 (yes), where higher scores indicate more autism traits. The mean of the scale was used and Cronbach’s alpha was 0.83 (questionnaire data) and 0.84 (experimental task data).

Caregiver ratings of conduct problems were measured by a questionnaire of 11 items, targeting the frequency of events that align with the diagnostic criteria of conduct disorder in the DSM-5, namely aggression towards people and animals, destruction of property, deceitfulness or theft and serious violations of rules (American Psychiatric Association, 2013). Items are rated on a scale from 1 (never), 2 (once), 3 (2–4 times), 4 (5–10 times) or 5 (more than 10 times), where higher scores indicate greater frequency of conduct problems. The mean of the scale was used and Cronbach’s alpha was 0.66 (questionnaire data) and 0.63 (experimental task data).

#### Socioeconomic status

Caregiver reports of SES was calculated by summing the score for each caregiver’s education level (0 = no university degree, 1 = university degree) and occupational status (0 = unemployed/seeking employment, working or studying 50% or less, in adapted employment or none of the options, 1 = working or studying more than 50%,), where the total score ranged from 0 to 4.

### Data analyses

#### Inter-rater reliability

For the experimental task, two assessors underwent training on the predefined emotion regulation strategies and their corresponding definitions. A total of 15 participants, with each recording containing 14 photo trials, were used for the training. Difficulties in interpreting verbal descriptions were resolved through discussion with the first author to ensure consensus. The prevalence-adjusted bias-adjusted kappa (PABAK) (Byrt et al., 1993) was used due to skewed response proportions of the use of regulation strategies. Final estimates revealed PABAK values of ≥ 0.77 for cognitive reappraisal, refocus on planning, putting into perspective, positive reappraisal, catastrophizing and suppression, suggesting at least substantial agreement (Landis & Koch, 1977). Percentual agreement was used for strategies with limited response variation of one or both raters. There was a 100% agreement for self-blame, other blame, acceptance, positive refocusing and rumination. See Supplementary Information for the full procedure of estimating inter-rater reliability.

#### Data processing

Complete missing data for three caregiver ratings in the questionnaire data, and for 13 caregivers not reporting co-occurring psychiatric symptoms in the experimental task data, were excluded in the analyses by pairwise deletion and listwise deletion, respectively. Additional missing data included one item for one caregiver on the SCAS-P (in both data sets) and 0.28% on emotional reactivity ratings during the experimental task. The latter was due to early withdrawal (n=1), difficulties interpreting photo content (n=2) and technical display issues (n=2). Missing data was not imputed given the relatively low proportion of missingness. For seven participants in the experimental data, voice recordings were incomplete or missing, mainly due to procedural errors. Here, emotional reactivity ratings and strategy use were based on notes from the physical test protocol.

#### Statistical analysis

Data were analysed using SPSS version 28. Differences regarding arousal (i.e., degree of emotional intensity) and valence (i.e., degree of pleasantness) levels, as reported by the IAPS manual (Lang et al., 2008), between the stimulus sets, as well as group differences on demographics and emotional reactivity (i.e., rated emotional distress), were assessed using independent sample t-tests or Mann-Whitney U tests (if the normality assumption was violated using the standardized skewness ±1.96) for continuous outcomes and χ2 tests for categorical outcomes. For the analyses, the independent variables were dichotomous (ADHD diagnosis [0,1], assigned sex at birth [0,1]) and continuous (age, SES). Self-rated use of regulation strategies was defined as continuous data and task-specific use of regulation strategies was treated as count data. For the main analyses, one outlier was detected for maladaptive strategies in the questionnaire data with a studentized residual of 3.05. In the experimental task data, three outliers were found for adaptive strategies, and four for maladaptive strategies, with standardized Pearson residuals of > 3. Further inspection revealed no measurement or processing errors, and Cook’s distance and leverage values were below threshold values (< 1 and < 0.2 respectively), why the participants was kept in the analyses. Removal of the outliers had no effect on significance patterns for the main findings.

Linear regressions were used for the questionnaire data. Statistical assumptions were examined by independence of observations (i.e., Durbin-Watson values between 1.5–2.5), linearity, homoscedasticity of residuals (assessed through scatter plots of unstandardized predicted values and studentized residuals), no multicollinearity (i.e., correlations of independent variables were <0.7 and variance inflation factors were ≤10) and normality (assessed through histograms and P-P plots of standardized residuals, as well as Q-Q plots of studentized residuals). For the experimental task data, generalized linear models (GLMs) were used with negative binomial distribution with log link. The model was chosen based on assessment of goodness-of-fit, which included comparing Pearson’s χ² statistic and Akaike information criteria values between negative binomial, Poisson and linear models.

The main analyses were conducted in separate models for each data set. First, we investigated the associations between ADHD and adaptive and maladaptive regulation strategies, while adjusting for sex, age and SES. Second, we additionally adjusted for co-occurring psychiatric symptoms. A Benjamini-Hochberg correction at a false discovery rate of 5% was used to adjust for multiple comparisons. Interaction effects (sex by diagnosis, and age by diagnosis) were examined using GLMs. Post-hoc tests included investigating associations between ADHD and self-rated use of specific regulation strategies using linear regressions. Adjustments were made for sex, age, SES, co-occurring psychiatric symptoms and multiple comparisons. Due to limited variability for the task-specific use of distinct regulation strategies, the dependent variables were coded as binary variables (0 = strategy not used, 1 = strategy not used) and analysed with logistic regressions. First, associations between ADHD and distinct regulation strategies were analysed. Next, adjustments were made for sex, age and SES, as well as multiple comparisons, but not co-occurring psychiatric symptoms due to low statistical power. See Supplementary Table 3 for frequencies of distinct strategy use for task-specific cognitive regulation. Two tailed tests with *p* < .05 were considered statistically significant.

## Results

### Group comparisons prior to the main analyses

#### Demographics

See Table 1 for descriptive statistics of sample characteristics. No age differences between groups (those with and without ADHD) were found for the questionnaire or experimental task data. Significant differences between the groups regarding sex were detected in the questionnaire data, but not in the experimental task data. The groups differed in SES in both the questionnaire data and experimental task.

#### Stimulus sets and descriptive statistics

See Tables 1 and 2 for descriptive statistics. There were no significant differences between set A and set B of the experimental task regarding mean valence, *t*(26) = 0.012, *p* = .990), or arousal, *t*(26) = -0.313, *p* = .757, based on the IAPS manual. The ADHD group and the comparison group did not differ in regards to self-ratings of emotional reactivity (emotional distress) during the experimental task. However, the ADHD group rated fewer adaptive strategies, more maladaptive strategies, less acceptance and more self-blame. In the experimental data, the ADHD group used less adaptive strategies and less putting into perspective. Mean emotional reactivity had a trend-level association with adaptive strategies (ρ = -0.15, *p* = .051), and were significantly related to maladaptive strategies (ρ = 0.25, *p* < .001).

**Table 2:** Descriptive statistics.

	*Questionnaire data*			*Experimental task data*		
	ADHD n=53	Comparison group n=41	Statistics	ADHD n=82	Comparison group n=94	Statistics
Adaptive strategies, m (SD)	2.54 (0.71)	2.89 (0.76)	t=2.30*	1.72 (2.33)	2.97 (3.15)	Z=2.83^⁎⁎^ Mdn: 1.00^a^ / 2.00^b^ IQR: 3.00^a^ / 5.25^b^
Maladaptive strategies, m (SD)	2.60 (0.66)	2.30 (0.52)	Z=2.23* Mdn: 2.50^a^ / 2.25^b^ IQR: 1.13^a^ / 0.75^b^	0.48 (0.76)	0.68 (1.31)	Z=0.11 Mdn: 0^a^ / 0^b^ IQR: 1.00^a^ / 1.00^b^
	M (SD)	M (SD)		n (%)	n (%)	
Specific regulation strategies						
Positive refocusing	2.14 (1.03)	2.48 (1.01)	Z=1.75 Mdn: 2.00^a^ / 2.50^b^ IQR: 1.75^a^ / 1.00^b^	0 (0.0)	1 (1.1)	χ2=0.88
Planning	2.55 (1.11)	2.94 (0.99)	t=1.77	22 (26.8)	29 (30.9)	χ2=0.34
Positive reappraisal	2.63 (1.12)	2.83 (1.17)	t=0.83	1 (1.2)	6 (6.4)	χ2=3.06
Putting into perspective	2.62 (1.19)	2.74 (1.09)	Z=0.76 Mdn: 2.00^a^ / 2.50^b^ IQR: 2.00^a^ / 1.50^b^	34 (41.5)	57 (60.6)	χ2=6.45*
Acceptance	2.76 (1.20)	3.48 (0.94)	t=3.24^⁎⁎^	4 (4.9)	8 (8.5)	χ2=0.91
Self-blame	2.61 (1.01)	2.09 (0.59)	Z=2.03* Mdn: 2.50^a^ / 2.00^b^ IQR: 2.00^a^ / 0.25^b^	3 (3.7)	2 (2.1)	χ2=0.37
Other-blame	2.35 (0.97)	2.21 (0.80)	Z=0.84 Mdn: 2.00^a^ / 2.00^b^ IQR: 1.00^a^ / 0.50^b^	2 (2.4)	6 (6.4)	χ2=1.57
Rumination	3.05 (1.03)	2.89 (0.85)	t=0.79	0 (0.0)	0 (0.0)	-
Catastrophizing	2.38 (1.05)	2.01 (0.78)	Z=1.48 Mdn: 2.00^a^ / 2.00^b^ IQR: 2.00^a^ / 1.00^b^	24 (29.3)	25 (26.6)	χ2=0.16
Cognitive reappraisal				22 (26.8)	30 (31.9)	χ2=0.54
Thought suppression				0 (0.0)	2 (2.1)	χ2=1.77
Other psychiatric symptoms						
Depressive symptoms c, m (SD)	1.29 (0.83)	.32 (0.43)	Z=6.08^⁎⁎⁎^ Mdn: 1.11^a^ / 0.22^b^ IQR: 1.14^a^ / 0.50^b^	1.26 (0.81)	.35 (0.44)	Z=7.82>⁎⁎⁎> Mdn: 1.11^a^ / 0.22^b^ IQR: 1.22^a^ / 0.56 b
Anxiety symptoms d, m (SD)	.68 (0.44)	.24 (0.15)	Z=5.61^⁎⁎⁎^ Mdn: 0.55^a^ / 0.24^b^ IQR: 0.64^a^ / 0.24^b^	.66 (0.41)	.27 (0.16)	Z=6.98^⁎⁎⁎^ Mdn: 0.54^a^ / 0.26^b^ IQR: 0.61^a^ / 0.20 b
Conduct problems e, m (SD)	1.38 (0.40)	1.05 (0.11)	Z=5.64^⁎⁎⁎^ Mdn: 1.36^a^ / 1.00^b^ IQR: 0.41^a^ / 0.09^b^	1.35 (0.38)	1.07 (0.12)	Z=6.54^⁎⁎⁎^ Mdn: 1.27^a^ / 1.00^b^ IQR: 0.36^a^ / 0.09 b
Autism traits f, m (SD)	.22 (0.15)	.06 (0.06)	Z=6.20^⁎⁎⁎^ Mdn: 0.18^a^ / 0.06^b^ IQR: 0.13^a^ / 0.10^b^	.24 (0.15)	.07 (0.07)	Z=8.61>^⁎⁎⁎^ Mdn: 0.20^a^ / 06^b^ IQR: 0.15^a^ / 06 b

*Note:* ADHD: Attention deficit/hyperactivity disorder; Mdn: median; IQR: interquartile range; ^⁎^: p <0.05⁎⁎ : p <0.01 ⁎⁎⁎: p <0.001

Three caregiver ratings for the questionnaire data, and 13 caregiver ratings (four were missing for adolescents with ADHD and nine for adolescents in the comparison group) on other psychiatric symptoms were excluded due to complete missing data

a Median/IQR for the ADHD group

b Median/IQR for the comparison group

c Measured by MADRS-P

d Measured by SCAS-P

e Measured by questionnaire targeting conduct disorder

f Measured by the Autism-Tics, AD/HD and other Comorbidities inventory in questionnaire form

### Associations between ADHD and self-rated cognitive emotion regulation

See Table 3 for the results. ADHD was associated with lower use of self-rated adaptive strategies and greater use of maladaptive strategies, accounting for sex, age and SES. However, the association with adaptive strategies was no longer significant after adjusting for multiple comparisons. In the second model, adjusting for co-occurring psychiatric traits, ADHD was no longer linked to usage of adaptive or maladaptive strategies. Girls reported using adaptive strategies less frequently, and maladaptive strategies more frequently, than boys. However, only the association with adaptive strategies remained in the fully adjusted model. No interaction effects of sex by diagnosis, nor age by diagnosis, were identified. Older age was associated with greater use of maladaptive strategies. Depressive symptoms were robustly linked to greater use of maladaptive strategies.

**Table 3:** Self-rated cognitive emotion regulation.

	Adaptive strategies				Maladaptive strategies			
	B (SE B)	β	p	R^2^_adj_	B (SE B)	β	p	R^2^_adj_
*Model 1*				.15				.19
ADHD	-32 (0.16)	-0.21	.044		.33 (0.13)	.27	**.010**	
Sex (female)	-0.54 (0.16)	-0.35	**< 0.001**		.32 (0.13)	.25	.012	
Age	-0.00 (0.05)	-0.00	.991		.14 (0.04)	.33	**< 0.001**	
SES	-0.16 (0.08)	-0.21	.045		.09 (0.06)	.15	.157	
*Model 2*				.12				.29
ADHD	-0.28 (0.23)	-0.18	.223		.05 (0.17)	.04	.758	
Sex (female)	-0.54 (0.18)	-0.35	**.003**		.19 (0.13)	.15	.150	
Age	.01 (0.05)	.01	.929		.13 (0.04)	.31	**.001**	
SES	-0.18 (0.09)	-0.23	.043		.11 (0.06)	.18	.083	
Depressive symptoms a	-0.03 (0.13)	-0.04	.805		.33 (0.10)	.45	**<0.001**	
Anxiety symptoms b	.01 (0.27)	.00	.980		.02 (0.20)	.01	.933	
Conduct problems c	-0.14 (0.27)	-0.07	.592		-0.07 (0.20)	-0.04	.737	
Autism traits d	.16 (0.69)	.03	.819		.05 (0.51)	.01	.916	

*Note*: ADHD: Attention deficit/hyperactivity disorder, SES: socioeconomic status. Bold indicates significant value after Benjamini-Hochberg correction. Three caregiver ratings were excluded by pairwise deletion for SES and co-occurring psychiatric symptoms

a Measured by MADRS-P

b Measured by SCAS-P

c Measured by questionnaire targeting conduct disorder

d Measured by the Autism-Tics, AD/HD and other Comorbidities inventory in questionnaire form

### Associations between task-specific cognitive emotion regulation

See Table 4 for the results. ADHD was linked to lower use of adaptive strategies (46% less frequent use compared to typically developing controls) during the experimental task while accounting for sex, age, SES and co-occurring psychiatric symptoms. However, the association was not significant after adjusting for multiple comparisons. Sex and age were not associated with any of the outcomes, and no significant interaction effects between sex and diagnosis, or age by diagnosis, were found.

**Table 4:** Task-specific cognitive emotion regulation.

	Adaptive strategies				Maladaptive strategies			
	b (SE b)	β	*p*	Exp (B)	b (SE b)	β	*p*	Exp (B)
*Model 1*								
ADHD	-0.49 (0.20)	-0.09	.015	.61	-0.23 (0.29)	-0.11	.412	.79
Sex (female)	-0.04 (0.19)	-0.06	.852	.97	-0.14 (0.26)	-0.06	.592	.87
Age	.05 (0.04)	.04	.287	1.05	-0.02 (0.06)	-0.04	.788	.98
SES	.20 (0.11)	.07	.065	1.22	.13 (0.15)	.12	.383	1.14
*Model 2*								
ADHD	-0.61 (0.29)	-0.11	.038	.54	-0.27 (0.40)	-0.12	.496	.76
Sex (female)	.07 (0.22)	.01	.761	1.07	-0.11 (0.28)	-0.05	.686	.89
Age	.07 (0.04)	.06	.140	1.07	-0.01 (0.06)	-0.02	.820	.99
SES	.24 (0.11)	.08	.039	1.27	.14 (0.16)	.13	.365	1.15
Depressive symptoms a	.05 (0.19)	.01	.778	1.05	.00 (0.25)	.00	.993	1.00
Anxiety symptoms b	-0.42 (0.43)	-0.05	.322	.66	-0.12 (0.51)	-0.04	.819	.89
Conduct problems c	-0.58 (0.45)	-0.06	.216	.57	.01 (0.54)	.00	.918	1.06
Autism traits d	2.05 (0.96)	.10	.032	7.75	.48 (1.33)	.06	.719	1.61

*Note*: ADHD: Attention deficit/hyperactivity disorder, SES: socioeconomic status. No associations were significant after Benjamini-Hochberg correction. A total of 13 participants were excluded by listwise deletion for missing data on caregiver ratings on SES and co-occurring psychiatric symptoms

a Measured by MADRS-P

b Measured by SCAS-P

c Measured by questionnaire targeting conduct disorder

d Measured by the Autism-Tics, AD/HD and other Comorbidities inventory in questionnaire form

### Post-hoc analyses

#### Distinct emotion regulation strategies

See Supplementary Table 4–7 for associations with specific regulation strategies. In the questionnaire data, ADHD was significantly associated with less use of positive refocusing and acceptance, and greater use of self-blame and catastrophizing. However, only the association between ADHD and less use of acceptance remained in the fully adjusted model. Girls used less positive reappraisal and acceptance, and more rumination and catastrophizing, compared to boys, albeit only the association to acceptance remained after all adjustments. Older age was associated with greater use of rumination, self-blame and catastrophizing, albeit the association with the latter was lost in the fully adjusted model. Depressive symptoms were linked to greater use of self-blame, catastrophizing and rumination, although the latter was no longer significant when adjusting for multiple comparisons.

In the experimental task data, ADHD was linked to lower use of putting into perspective (54% less frequent use compared to typically developing controls) although the association did not remain after adjusting for sex, age and SES. No significant sex- or age-related patterns were identified. Note that some strategies (positive refocusing, rumination and thought suppression) could not be modelled correctly due to limited variability, whereas other strategies (positive reappraisal, self-blame and other-blame) had fewer than 10 cases where frequency use was 1 or above.

#### Associations with ADHD symptoms

As a post-hoc analysis, caregiver-rated ADHD symptoms, assessed using the World Health Organization Adult ADHD Self-Report Scale for adolescents (ASRS-AP) (Sjölander et al., 2016), replaced clinical diagnosis as the independent variable in the main analyses. Results were largely similar, except that ADHD symptoms were not significantly associated with self-rated or task-specific use of adaptive strategies (*p*s = >0.05).

## Discussion

We examined self-rated and task-specific use of cognitive emotion regulation strategies in children and adolescents with ADHD, while also accounting for potential sex and age effects, as well as co-occurring psychiatric symptoms. To the best of our knowledge, this has previously not been examined, thereby contributing greater specificity to the field. We found that ADHD was associated with a lower self-rated and task-specific use of adaptive strategies, and a greater self-rated use of maladaptive strategies. However, no associations remained when adjusting for co-occurring psychiatric symptoms and multiple comparisons. Therefore, we cannot exclude the possibility that the effects are better explained by factors shared between ADHD and other forms of psychopathology. Post-hoc analyses revealed a robust link between ADHD and less self-rated use of acceptance. No sex-specificity in the association between ADHD and emotion regulation was found, although girls reported using adaptive strategies less frequently.

For rated cognitive emotion regulation, some studies suggest that greater use of maladaptive strategies is characteristic for ADHD (Alacha et al., 2025; Rüfenacht et al., 2019). However, the studies did not account for co-occurring psychiatric traits and our findings indicate that depressive symptoms may better explain this association. This aligns with previous research linking depressive symptoms to greater rated use of maladaptive strategies (Mayer et al., 2022; Nolen-Hoeksema & Aldao, 2011). Conversely, Mayer and colleagues (2022) found that individuals with ADHD used maladaptive strategies more often than controls, even when excluding participants with co-occurring depression. Notably, the authors adjusted for depression using diagnoses, which may mask potential effects of subthreshold cases, namely individuals who may not fulfil the diagnostic criteria but still experience depressive symptoms. Corroborating this, Mayer and colleagues (2022) found that depressive, not ADHD, symptoms were linked to greater self-rated use of maladaptive strategies, as well as lower self-rated use of adaptive strategies. This is consistent with our results, suggesting that co-occurring depressive symptoms may play a more vital role than ADHD for explaining maladaptive strategy use. Further, this underscores the need to consider co-occurring psychiatric traits when investigating emotion regulation in ADHD.

For task-specific cognitive emotion regulation, ADHD was only linked to lower use of adaptive strategies, and not to the use of maladaptive strategies, although this association did not remain after adjusting for multiple comparisons. The null finding for maladaptive strategies may have been particularly affected by their low frequency of use, where for example rumination was not used by a single participant. This could reflect difficulties to fully capture maladaptive strategies through verbal descriptions, especially in an experimental setting. For example, participants may be less willing to share negative cognitions, such as blaming oneself or others, with a test leader. Further, such reactions may not be evoked by merely viewing photos. Brief descriptions may also fail to capture specific strategies, such as rumination, as they may unfold over time. Moreover, ratings of emotional reactivity (in this study, emotional distress) did not differ between the groups in our sample, even though previous research suggests that ADHD is linked to increased emotional reactivity (Graziano & Garcia, 2016). As Hagstrøm and colleagues (2020) argue, it is possible that the emotional stimuli did not elicit a strong enough of a response, which in our study may have reduced the need for using regulation strategies in general. If stronger emotional reactions were evoked, differences between the groups may become more apparent. Thus, investigating task-specific cognitive emotion regulation during experimental tasks may benefit from using more ecologically valid stimuli to induce stronger emotional reactions and investigating the temporal dynamics of using regulation strategies over a broader time frame.

Albeit no associations between ADHD and self-rated and task-specific cognitive emotion regulation remained significant after all adjustments in the main analyses, some notable findings emerged in the post-hoc analyses. Specifically, we found that ADHD was robustly associated with a lower self-rated use of acceptance, with the same direction in task-specific use of acceptance, although this was not significant. Previous research suggest that acceptance is frequently used during adolescence in the general population and has been shown to be an effective regulation strategy (Lennarz et al., 2019), linked to less psychopathology (Aldao & Nolen-Hoeksema, 2012) and daily positive affect (Boemo et al., 2022). Thus, a lower use of acceptance could be an important factor for understanding emotional challenges in child- and adolescent ADHD. There was also a significant association between lower task-specific use of putting in perspective and ADHD, suggesting that this distinct strategy may also be relevant for emotion regulation in ADHD. Albeit the association did not remain after adjusting for sex, age and SES, this could be explained by low statistical power and should be investigated further. We did not find any association between ADHD and the task-specific use of cognitive reappraisal, which is in line with previous studies showing that individuals with ADHD perform comparably to controls when guided in the use of cognitive reappraisal (e.g., Hagstrøm et al., 2020; Kiani et al., 2024; Van Cauwenberge et al., 2017). However, the overall task-specific use of strategies was low, why findings need to be interpreted with caution.

Regarding sex- and age-effects, the most robust findings in our study suggests that girls report using adaptive strategies, especially acceptance, less frequently in daily life compared to boys, consistent with recent research of adolescents and young adults in the general population (Smith et al., 2023). Even though girls also reported using more maladaptive strategies than boys, aligning with previous research (Nolen-Hoeksema & Aldao, 2011; Smith et al., 2023; Zimmermann & Iwanski, 2014), our findings suggest that this effect may be driven by co-occurring depressive symptoms. Post-hoc analyses further revealed that the role of co-occurring depressive symptoms may be particularly pronounced for catastrophizing, and possibly rumination, albeit findings were not as robust for the latter. In contrast, Nolen-Hoeksema & Aldao (2011) found that adult women use rumination more than men, even when adjusting for co-occurring depressive symptoms. This discrepancy may reflect a developmental difference, where depressive symptoms could be a stronger predictor of maladaptive strategy use for girls during adolescence compared to adulthood, which necessitates further investigation. Further, older participants had higher self-rated use of maladaptive strategies than their younger counterparts, which may partly be driven by increased use of self-blame and rumination. Regarding the latter, it has been suggested that the use of rumination decreases with age, at least during adulthood (Nolen-Hoeksema & Aldao, 2011), which may be in contrast to our findings. Together, this may indicate that the use of rumination may peak during late adolescence before declining more gradually across adulthood. However, more longitudinal research is needed to explore possible developmental specificity in the use of emotion regulation strategies.

Findings need to be considered in the context of the strengths and limitations of the study. Limitations include methodological concerns in assessing task-specific cognitive emotion regulation, including low strategy use reducing statistical power and hindering the ability to analyse specific strategies (e.g., rumination), as well as the lack of group differences in emotional reactivity to the photos in the experimental task. Regarding the latter, it may be fruitful to use more dynamic emotion-eliciting stimuli (e.g., using video clips or virtual reality) to increase ecological validity (Sonkusare et al., 2019). We did not include an objective measure of visual attention to confirm that participants consistently viewed the emotional images as instructed. Although the experimenter monitored task compliance, we cannot entirely exclude the possibility that occasional attentional lapses or avoidance behaviours (e.g., looking away from aversive stimuli or talkativeness) occurred. This may be particularly relevant for children with ADHD, who are more prone to distractibility or variable task engagement. Although the emotion-eliciting photos were selected based on normative IAPS ratings for valence and arousal, these values may not fully reflect participants’ subjective experiences. In addition, valence and arousal were not entered as covariates in the analyses, which could influence the specific regulation strategies elicited by each image.

Self-rated cognitive emotion regulation was assessed with CERQ-short, which assumes respondents can accurately reflect on their cognitive processes. Further, regarding distinct emotion regulation strategies, in the CERQ-short, each strategy is assessed with only two items, affecting the internal consistency. In our sample, Cronbach alphas were good for adaptive strategies, but questionable for maladaptive strategies. Unidirectional wording which is used in CERQ-short can inflate correlations, increase response bias, and reduce the reliability and validity of the scale. To complement subjective ratings for emotional intensity, more objective measures may be used. For instance, emotional processing in ADHD has been linked to atypical patterns of autonomic reactivity or arousal (Bellato et al., 2020), which may be investigated through methods such as pupillometry (Bradley et al., 2008) or heart-rate variability (Bunford et al., 2017).

For the questionnaire data, 71.7% in the ADHD group were girls, which does not represent the typical sex ratio in the ADHD population (American Psychiatric Association, 2013) and may possibly stem from self-selection bias in this population, where females with ADHD may be more willing than their male peers to participate in research projects (Meyer et al., 2022), hence threatening the generalizability of results. For instance, females with ADHD are often under-diagnosed compared to males (Beheshti et al., 2020; Bruchmüller et al., 2012), and tends to present with more inattentive symptoms (American Psychiatric Association, 2013). This should be considered when interpreting our findings, as differences in symptom presentation may affect both self-reported emotion regulation strategies and the relationship between ADHD and emotional functioning (Faraone et al., 2019). Accordingly, in our samples, approximately 40% of those with ADHD had a predominantly inattentive presentation. Even though sex was adjusted for across all models, future studies should aim to balance the sex ratio of the participants.

Caregivers in the ADHD group had lower rated SES compared to caregivers in the control group, however, this is to be expected since young individuals with ADHD in Sweden has been reported to be more likely to have more social adversities and parents with lower education (Björkenstam et al., 2018). Caregiver reports of co-occurring psychiatric symptoms may not fully represent the participants’ experiences, particularly internalizing symptoms, which are often less observable to others. We used caregiver education and occupation as a proxy for SES, which provides only a broad estimate and may not fully capture other important aspects such as wealth or neighbourhood characteristics, potentially limiting the precision of SES-related analyses.

Strengths include assessing cognitive emotion regulation in two distinct aspects, namely self-rated use and task-specific use, and including a diagnostic cohort that has been thoroughly assessed. Further, adjustments for sex, age and co-occurring psychiatric symptoms (i.e., factors that may be influential for emotion regulation) were made to increase specificity.

## Conclusions

This study expands our understanding of cognitive emotion regulation in child- and adolescent ADHD. Findings elucidate that the associations between ADHD and cognitive emotion regulation may be less robust when considering other influential factors, such as co-occurring psychiatric symptoms. Specifically, depressive symptoms may be of particular interest when it comes to accounting for self-rated use of maladaptive strategies. Further, post-hoc analyses indicated that reduced self-rated use of acceptance, and possibly putting in perspective, may contribute to emotion challenges in ADHD. Sex-related effects included girls using fewer adaptive strategies than boys, and more maladaptive strategies, albeit the latter seems to be driven by depressive symptoms. The use of self-rated maladaptive strategies may increase over time, though this development may follow a non-linear trajectory, with adolescence potentially representing a sensitive period for such changes.

## Consent for publication

All participants and caregivers gave consent for publication.

## Availability of data and materials

Consent for sharing individual data outside the research team was not obtained. However, reasonable requests for patient-level data can be made to the corresponding author and will be considered after discussion with the ethical review board. Relevant data are included in the manuscript or supplementary information files.

## Declaration of generative AI and AI-assisted technologies in the writing process

During the preparation of this work the author(s) used ChatGPT 4.o sparingly in order to improve readability and language. After using this tool/service, the author(s) reviewed and edited the content as needed and take(s) full responsibility for the content of the published article.

## Funding

Open access funding was provided by Uppsala University. Funding for this study was provided by the Foundation Sunnerdahl’s Disability Fund (F10/21 and F19/22), the Swedish Brain Foundation (FO2020–0128), the Uppsala University Hospital Research Fund (ALF), Uppsala County Council’s Funds for Clinical Research ((LUL-977,413; LUL-1001,951), Regional Research Council Mid Sweden (RFR-980,939), The Foundation in Memory of Professor Bror Gadelius and Uppsala University (Medicinska Fakultetens Stiftelse för Psykiatrisk och Neurologisk Forskning). The funders had no influence over the design, data collection, analysis, interpretation or in writing the manuscript of this study.

## Declaration of competing interest

The authors declare the following financial interests/personal relationships which may be considered as potential competing interests:

Johan Isaksson reports financial support was provided by Sunnerdahls Foundation Disability Fund. Johan Isaksson reports financial support was provided by Swedish Brain Foundation. Johan Isaksson reports financial support was provided by Uppsala University Hospital Research Fund (ALF). Johan Isaksson reports financial support was provided by Regional Research Council Mid Sweden. Rebecka Astenvald reports financial support was provided by Foundation Professor Bror Gadelius Memorial Fund. Rebecka Astenvald reports financial support was provided by Uppsala University (Medicinska fakultetens stiftelse for Psykiatrisk och Neurologisk Forskning). Rebecka Astenvald reports financial support was provided by Region Uppsala. If there are other authors, they declare that they have no known competing financial interests or personal relationships that could have appeared to influence the work reported in this paper.

## Appendix Supplementary materials

Image, application 1
