# Supplementary material for: Adaptive and maladaptive cognitive emotion regulation in child- and adolescent ADHD
Source: Int J Clin Health Psychol. 2026 Jan 8;26(1):100660. doi: 10.1016/j.ijchp.2025.100660 (PMC12813563; doi:10.1016/j.ijchp.2025.100660)
Supplement: Supplementary file 1 [file mmc1.docx]

Supplementary information

**Table of contents**

Training examples for coding statements of verbal descriptions (Supplementary Table 1)

Estimations of interrater-reliability (including Supplementary Table 2)

Frequency use of state-level cognitive emotion regulation strategies (Supplementary Table 3)

Post-hoc analyses (Supplementary Table 4–7)

| Supplementary Table 1. Training examples for coding statements across each regulation strategy during the experimental task | |
| --- | --- |
|  | Training examples |
| *Adaptive strategies* |  |
| Positive refocusing | “I started thinking about [pleasant or nice experience] instead”; “I went to my happy place” |
| Planning | “I think about how I can change this situation”; “I would call an ambulance” |
| Positive reappraisal | “I think that the situation also has positive sides to it”; “I think I/they can learn something from this” |
| Putting into perspective | “It could have been much worse”; “This is not very likely to happen” |
| Acceptance | “I think I have to learn how to live with experiencing this”; “There is nothing I can do to change this situation” |
| Cognitive reappraisal | “It’s just a photo of the accident, it is not real”; “Everyone will survive since the rescue team is on their way” |
|  |  |
| *Maladaptive strategies* |  |
| Self-blame | “I would think that this is my fault”; “I think about mistakes I have made like this” |
| Other-blame | “This is their own fault”; “He/she is responsible for causing a scene like this” |
| Rumination | “I can’t stop thinking about how horrible this must have been”; “I can’t really let it [the image] go” |
| Catastrophizing | “It’s not possible for them to make it, no one will survive”; “This must be the worst possible thing to happen” |
| Thought suppression | “I would think about something else”; “I would try my best not to think about it” |

**Inter-rater reliability procedure**

A negative response (0) indicated that the specific emotion regulation strategy was not used, while a positive response (1) indicated that it was used. First, kappa values were calculated based on 10 participants, with 14 trials each, for all strategies. After the initial coding, strategies were reassessed with additional data (up to five participants) due to challenges in interpreting verbal descriptions (i.e., a qualitative judgment was made that the assessors were not adequately trained to assess specific strategies) and/or because of limited response variability hindering calculations of Kappa values. Before rating the additional data, assessors underwent further training to ensure accurate and consistent ratings. Final inter-rater reliability estimates of the initial and additional data is presented below, in Supplementary Table 2. If limited variability still hindered the calculations of Kappa values after the reassessments, percentual agreement was used as an alternative measure of inter-rater reliability.

| Supplementary Table 2. Inter-rater reliability estimates | | | |
| --- | --- | --- | --- |
|  | Percentual agreement ^a^ | PABAK ^b^ | Cohen’s kappa |
| *Strategy* |  |  |  |
| Cognitive reappraisal | 94.2% ^c^, 88.6% ^d^ | 0.89 ^c^, 0.77 ^d^ | 0.18 ^c^, 0.77 ^d^ |
| Acceptance | 99.3% ^c^, 100% ^d^ |  |  |
| Positive refocusing | 99.3% ^c^, 100% ^d^ |  |  |
| Refocus on planning | 98.6% ^c^, 95.7% ^d^ | 0.97 ^c^, 0.91 ^d^ | 0.79 ^c^, 0.71 ^d^ |
| Positive reappraisal | 100% ^c^, 100% ^d^ | 1.00 ^d^ | 1.00 ^d^ |
| Putting into perspective | 95.0% ^c^, 92.8% ^d^ | 0.90 ^c^, 0.857 ^d^ | 0.61 ^c^,0.74 ^d^ |
| Self-blame | 100% ^c^, 100% ^d^ |  |  |
| Rumination | 98.6% ^c^, 100% ^d^ | 0.97 ^c^ | 0.00 ^c^ |
| Catastrophizing | 86.4% ^c^, 98.6% ^d^ | 0.73 ^c^, 0.97 ^d^ | 0.00 ^c^, 0.66 ^d^ |
| Other-blame | 100% ^c^, 100% ^d^ |  |  |
| Suppression | 100% ^c^, 92.9% ^d^ | 0.86 ^d^ | 0.63 ^d^ |
| *Note*: Kappa values could not be calculated for acceptance, positive refocusing, self-blame and other-blame due to limited variability of responses (negative responses constant).  a = Percentual agreement based on the number of positive and negative responses that both raters agreed upon divided by the total amount of responses (a+d/N)  b = Prevalence-adjusted bias-adjusted kappa (PABAK)  c = Based on initial data of 10 participants with 14 trials each  d = Based on additional data of up to five participants with 14 trials each | | | |

| Supplementary Table 3. Frequency use of state-level cognitive emotion regulation strategies across the entire experimental task data set (n=176) | |
| --- | --- |
|  | Used (1)  n (%) |
| *Adaptive strategies* |  |
| Positive refocusing | 1 (0.6%) |
| Planning | 51 (29.0%) |
| Positive reappraisal | 7 (4.0%) |
| Putting into perspective | 91 (51.7%) |
| Acceptance | 12 (6.8%) |
| Cognitive reappraisal | 52 (29.5%) |
|  |  |
| *Maladaptive strategies* |  |
| Self-blame | 5 (2.8%) |
| Other-blame | 8 (4.5%) |
| Rumination | 0 (0%) |
| Catastrophizing | 49 (27.8%) |
| Thought suppression | 2 (1.1%) |

| Supplementary Table 4. Prediction of distinct trait-level adaptive regulation strategies | | | | | | | | | | | | | | | | | | | | |
| --- | --- | --- | --- | --- | --- | --- | --- | --- | --- | --- | --- | --- | --- | --- | --- | --- | --- | --- | --- | --- |
|  | Positive refocusing | | | | Planning | | | | Positive reappraisal | | | | Putting into perspective | | | | Acceptance | | | |
|  | B (SE B) | β | *p* | R^2^ _adj_ | B (SE B) | β | *p* | R^2^ _adj_ | B (SE B) | β | *p* | R^2^ _adj_ | B (SE B) | β | *p* | R^2^ _adj_ | B (SE B) | β | *p* | R^2^ _adj_ |
| *Model 1* |  |  |  | .07 |  |  |  | .05 |  |  |  | .04 |  |  |  | -.00 |  |  |  | .36 |
| ADHD | -.46 (.23) | -.22 | .047 |  | -.20 (.24) | -.09 | .412 |  | -.13 (.26) | -.06 | .617 |  | -.16 (.26) | -.07 | .533 |  | -.67 (.21) | -.29 | .**002** |  |
| Sex (female) | -.32 (.22) | -.15 | .151 |  | -.34 (.24) | -.15 | .153 |  | -.56 (.25) | -.24 | .028 |  | -.32 (.26) | -.14 | .222 |  | -1.16 (.21) | -.50 | **<.001** |  |
| Age | -.14 (.07) | -.19 | .067 |  | .14 (.08) | .18 | .081 |  | .07 (.08) | .09 | .373 |  | -.11 (.08) | -.14 | .204 |  | .03 (.07) | .04 | .645 |  |
| SES | -.25 (.12) | -.23 | .036 |  | .11 (.12) | .10 | .372 |  | -.15 (.13) | -.13 | .244 |  | -.12 (.13) | -.11 | .353 |  | -.40 (.11) | -.34 | **<.001** |  |
|  |  |  |  |  |  |  |  |  |  |  |  |  |  |  |  |  |  |  |  |  |
| *Model 2* |  |  |  | .11 |  |  |  | .07 |  |  |  | -.00 |  |  |  | -.01 |  |  |  | .34 |
| ADHD | -.25 (.31) | -.12 | .425 |  | -.03 (.33) | -.01 | .928 |  | -.07 (.36) | -.03 | .856 |  | -.18 (.37) | -.08 | .617 |  | -.86 (.30) | -.38 | **.005** |  |
| Sex (female) | -.29 (.25) | -.14 | .245 |  | -.41 (.26) | -.19 | .122 |  | -.45 (.29) | -.19 | .122 |  | -.47 (.29) | -.20 | .110 |  | -1.09 (.24) | -.47 | **<.001** |  |
| Age | -.11 (.07) | -.15 | .141 |  | .13 (.08) | .18 | .096 |  | .07 (.08) | .09 | .398 |  | -.10 (.09) | -.12 | .264 |  | .03 (.07) | .03 | .710 |  |
| SES | -.30 (.12) | -.29 | .012 |  | .09 (.13) | .08 | .464 |  | -.14 (.14) | -.12 | .312 |  | -.16 (.14) | -.14 | .255 |  | -.37 (.11) | -.31 | **.002** |  |
| Depressive  symptoms ^a^ | -.31 (.18) | -.25 | .086 |  | .38 (.19) | .29 | .051 |  | .02 (.21) | .01 | .942 |  | -.20 (.21) | -.15 | .349 |  | -.04 (.17) | -.03 | .810 |  |
| Anxiety  symptoms ^b^ | .18 (.38) | .07 | .633 |  | -.52 (.40) | -.20 | .196 |  | -.39 (.44) | -.14 | .386 |  | .77 (.45) | .27 | .090 |  | .01 (.36) | .00 | .989 |  |
| Conduct  problems ^c^ | -.51 (.37) | -.17 | .171 |  | -.50 (.39) | -.16 | .205 |  | -.02 (.43) | -.01 | .969 |  | -.03 (.48) | -.01 | .954 |  | .34 (.35) | .10 | .343 |  |
| Autism traits ^d^ | .99 (.95) | .14 | .299 |  | -.89 (1.01) | -.12 | .377 |  | .52 (1.11) | .06 | .644 |  | -.62 (1.12) | -.08 | .584 |  | .78 (.91) | .10 | .390 |  |
| *Note*: ADHD: Attention deficit/hyperactivity disorder, SES: socioeconomic status. Bold indicates significant value after Benjamini-Hochberg correction. Three caregiver ratings were excluded by pairwise deletion for SES and co-occurring psychiatric symptoms  ^a^ Measured by MADRS-P  ^b^ Measured by SCAS-P  ^c^ Measured by questionnaire targeting conduct disorder  ^d^ Measured by the Autism-Tics, AD/HD and other Comorbidities inventory in questionnaire form | | | | | | | | | | | | | | | | | | | | |

| Supplementary Table 5. Prediction of distinct trait-level maladaptive regulation strategies | | | | | | | | | | | | | | | | | |
| --- | --- | --- | --- | --- | --- | --- | --- | --- | --- | --- | --- | --- | --- | --- | --- | --- | --- |
|  | Self-blame | | | | Other-blame | | | | Rumination | | | | Catastrophizing | | | | |
|  | B (SE B) | β | *p* | R^2^ _adj_ | B (SE B) | β | *p* | R^2^ _adj_ | B (SE B) | β | *p* | R^2^ _adj_ | B (SE B) | β | *p* | R^2^ _adj_ |  |
| *Model 1* |  |  |  | .17 |  |  |  | .01 |  |  |  | .15 |  |  |  | .14 |  |
| ADHD | .66 (.21) | .33 | **.002** |  | .04 (.20) | .02 | .863 |  | .23 (.20) | .12 | .264 |  | .42 (.20) | .22 | .041 |  |  |
| Sex (female) | .33 (.21) | .16 | .117 |  | -.13 (.20) | -.07 | .510 |  | .54 (.20) | .28 | **.008** |  | .54 (.20) | .28 | **.007** |  |  |
| Age | .23 (.07) | .33 | **.001** |  | -.04 (.07) | -.06 | .588 |  | .21 (.06) | .32 | **.002** |  | .16 (.06) | .25 | **.014** |  |  |
| SES | .20 (.11) | .19 | .070 |  | -.21 (.10) | -.23 | .046 |  | .19 (.10) | .19 | .068 |  | .19 (.10) | .20 | .063 |  |  |
|  |  |  |  |  |  |  |  |  |  |  |  |  |  |  |  |  |  |
| *Model 2* |  |  |  | .21 |  |  |  | -.03 |  |  |  | .21 |  |  |  | .23 |  |
| ADHD | .44 (.29) | .22 | .126 |  | -.01 (.29) | -.00 | .982 |  | -.17 (.27) | -.09 | .521 |  | -.06 (.27) | -.03 | .820 |  |  |
| Sex (female) | .27 (.23) | .13 | .241 |  | -.17 (.23) | -.09 | .462 |  | .33 (.22) | .17 | .134 |  | .34 (.21) | .18 | .111 |  |  |
| Age | .20 (.07) | .30 | **.003** |  | -.03 (.07) | -.05 | .628 |  | .20 (.06) | .31 | **.002** |  | .14 (.06) | .22 | .022 |  |  |
| SES | .24 (.11) | .23 | .033 |  | -.22 (.11) | -.24 | .053 |  | .20 (.10) | .20 | .061 |  | .23 (.10) | .24 | .026 |  |  |
| Depressive symptoms ^a^ | .44 (.17) | .37 | **.009** |  | .07 (.17) | .07 | .665 |  | .36 (.16) | .32 | .023 |  | .46 (.16) | .40 | **.004** |  |  |
| Anxiety symptoms ^b^ | -.37 (.35) | -.15 | .291 |  | -.00 (.35) | -.00 | .995 |  | .26 (.33) | .11 | .427 |  | .18 (.32) | .08 | .576 |  |  |
| Conduct problems ^c^ | .07 (.34) | .02 | .835 |  | -.19 (.35) | -.07 | .593 |  | -.24 (.32) | -.09 | .450 |  | .10 (.32) | .03 | .765 |  |  |
| Autism traits ^d^ | -.31 (.87) | -.04 | .721 |  | .24 (.89) | .04 | .787 |  | .40 (.82) | .06 | .630 |  | -.11 (.81) | -.02 | .892 |  |  |
| *Note*: ADHD: Attention deficit/hyperactivity disorder, SES: socioeconomic status. Bold indicates significant value after Benjamini-Hochberg correction. Three caregiver ratings were excluded by pairwise deletion for SES and co-occurring psychiatric symptoms  ^a^ Measured by MADRS-P  ^b^ Measured by SCAS-P  ^c^ Measured by questionnaire targeting conduct disorder  ^d^ Measured by the Autism-Tics, AD/HD and other Comorbidities inventory in questionnaire form | | | | | | | | | | | | | | | | |  |

| Supplementary Table 6. Prediction of distinct state-level adaptive regulation strategies | | | | | | | | | | | | | | | |  | | | |
| --- | --- | --- | --- | --- | --- | --- | --- | --- | --- | --- | --- | --- | --- | --- | --- | --- | --- | --- | --- |
|  | Positive refocusing^a^ | | | Planning | | | Positive reappraisal ^b^ | | | Putting into perspective | | | Acceptance | | | Cognitive reappraisal | | | |
|  | B (SE B) | *p* | *Exp (B)* | B (SE B) | *p* | *Exp (B)* | B (SE B) | *p* | *Exp (B)* | B (SE B) | *p* | *Exp (B)* | B (SE B) | *p* | *Exp (B)* | B (SE B) | *p* | *Exp (B)* |  |
| *Model 1* |  |  |  |  |  |  |  |  |  |  |  |  |  |  |  |  |  |  |  |
| ADHD |  |  |  | -.20 (.34) | .558 | 0.82 | -1.71 (1.09) | .117 | 0.18 | -.78 (.31) | **.012** | 0.46 | -.60 (.63) | .346 | 0.55 | -.25 (.33) | .461 | 0.78 |  |
|  |  |  |  |  |  |  |  |  |  |  |  |  |  |  |  |  |  |  |  |
| *Model 2* |  |  |  |  |  |  |  |  |  |  |  |  |  |  |  |  |  |  |  |
| ADHD |  |  |  | -.26 (.37) | .486 | 0.78 | -1.22 (1.15) | .290 | 0.30 | -.63 (.34) | .065 | 0.53 | -1.09 (.75) | .149 | 0.34 | .00 (.37) | 1.000 | 1.00 |  |
| Sex (female) |  |  |  | .35 (.36) | .326 | 1.14 | -1.84 (1.11) | .098 | 0.16 | -.39 (.33) | .245 | 0.68 | 1.25 (.76) | .101 | 3.47 | -.06 (.35) | .865 | 0.94 |  |
| Age |  |  |  | .07 (.08) | .361 | 1.07 | .15 (.18) | .398 | 1.16 | .05 (.07) | .475 | 1.05 | .32 (.16) | .043 | 1.37 | -.01 (.08) | .929 | 0.99 |  |
| SES |  |  |  | .06 (.19) | .751 | 1.06 | .50 (.69) | .471 | 1.64 | .37 (.18) | .039 | 1.45 | .60 (.47) | .201 | 1.83 | .28 (.20) | .162 | 1.32 |  |
| *Note*: ADHD: Attention deficit/hyperactivity disorder, SES: socioeconomic status. Bold indicates significant value after Benjamini-Hochberg correction. A total of 13 participants in the second model were excluded by listwise deletion for missing data on caregiver ratings of SES and co-occurring psychiatric symptoms  ^a^ Could not be modelled correctly due to highly limited variability of the specific strategy, why statistics are not reported  ^b^ The frequency of usage for the regulation strategy were less than 10 cases | | | | | | | | | | | | | | | | | | | |

| Supplementary Table 7. Prediction of distinct state-level maladaptive regulation strategies | | | | | | | | | | | | | | | |
| --- | --- | --- | --- | --- | --- | --- | --- | --- | --- | --- | --- | --- | --- | --- | --- |
|  | Self-blame ^b^ | | | Other-blame ^b^ | | | Rumination^a^ | | | Catastrophizing | | | Thought suppression^a^ | | |
|  | B (SE B) | *p* | *Exp (B)* | B (SE B) | *p* | *Exp (B)* | B (SE B) | *p* | *Exp (B)* | B (SE B) | *p* | *Exp (B)* | B (SE B) | *p* | *Exp (B)* |
| *Model 1* |  |  |  |  |  |  |  |  |  |  |  |  |  |  |  |
| ADHD | .56 (.93) | .547 | 1.75 | -1.00 (.83) | .227 | 0.37 |  |  |  | .13 (.34) | .693 | 1.14 |  |  |  |
|  |  |  |  |  |  |  |  |  |  |  |  |  |  |  |  |
| *Model 2* |  |  |  |  |  |  |  |  |  |  |  |  |  |  |  |
| ADHD | .22 (1.03) | .830 | 1.25 | -.55 (.87) | .525 | 0.58 |  |  |  | .22 (.38) | .561 | 1.24 |  |  |  |
| Sex (female) | .46 (.96) | .636 | 1.58 | -.69 (.77) | .368 | 0.50 |  |  |  | -.00 (.36) | .994 | 1.00 |  |  |  |
| Age | -.30 (.24) | .208 | 0.74 | -.25 (.19) | .195 | 0.78 |  |  |  | .08 (.08) | .286 | 1.09 |  |  |  |
| SES | -.50 (.46) | .282 | 0.61 | .94 (.66) | .155 | 2.55 |  |  |  | .09 (.19) | .654 | 1.09 |  |  |  |
| *Note*: ADHD: Attention deficit/hyperactivity disorder, SES: socioeconomic status. Bold indicates significant value after Benjamini-Hochberg correction. A total of 13 participants in the second model were excluded by listwise deletion for missing data on caregiver ratings of SES and co-occurring psychiatric symptoms  ^a^ Could not be modelled correctly due to highly limited variability of the specific strategy, why statistics are not reported ^b^ The frequency of usage for the regulation strategy were less than 10 cases | | | | | | | | | | | | | | | |
